# Public deliberation to assess patient views on biosimilar medication switching for the treatment of inflammatory bowel disease

**DOI:** 10.1186/s12913-024-11570-3

**Published:** 2024-10-09

**Authors:** Kerry A. Ryan, Shirley Cohen-Mekelburg, Jessica A. Baker, Eileen M. Weinheimer-Haus, Chris Krenz, Jason K. Hou, Raymond De Vries, Akbar K. Waljee

**Affiliations:** 1 Center for Bioethics and Social Sciences in Medicine, University of Michigan Medical School, Ann Arbor, MI USA; 2 Health Services Research and Development Center of Clinical Management Research, VHA Ann Arbor, Ann Arbor, MI USA; 3 Michigan Medicine, Department of Internal Medicine, Division of Gastroenterology and Hepatology, Ann Arbor, MI USA; 4 Michigan Medicine, Department of Learning Health Sciences, Ann Arbor, MI USA; 5 Center for Innovations in Quality, Effectiveness and Safety (IQuESt), Michael E. DeBakey, Veterans Affairs Medical Center, Houston, TX USA; 6 Department of Medicine, Section of Gastroenterology and Hepatology, Baylor College of Medicine, Houston, TX USA

**Keywords:** Patient-centered care, Inflammatory bowel disease, Biosimilar switching, Deliberative democracy, Patient preferences

## Abstract

**Background:**

Biosimilars are highly similar, but not identical, versions of originator biologic medications. Switching patients to biosimilars presents an opportunity to mitigate rising drug costs and expand patient access to important biologic therapies. However, decreased patient acceptance and adherence to biosimilar medications have been reported, which can lead to loss of treatment response, adverse reactions, and inefficient resource utilization. Understanding patient perceptions of biosimilars and biosimilar switching is needed to inform patient-centered care strategies that promote efficient resource utilization.

**Methods:**

We used democratic deliberation methods to solicit the informed and considered opinions of patients regarding biosimilar switching. Patients with inflammatory bowel disease (IBD; *n* = 29) from the Veterans Health Administration (VHA) participated in 5-hour deliberation sessions over two days. Following educational presentations with experts, participants engaged in facilitated small group discussions. Transcripts and facilitators’ notes were used to identify key themes. Participants completed surveys pre- and post-deliberation to collect sociodemographic and clinical features as well as to assess IBD treatment knowledge and attitudes toward care and approaches to biosimilar switching.

**Results:**

Five major themes emerged from the small group discussions in the context of biosimilar switching: 1) concerns about adverse consequences and unclear risk-benefit balance; (2) importance of communication and transparency; (3) desire for shared decision making and patient involvement in treatment decisions; (4) balancing cost-saving with competing priorities; and (5) advocating for individualized care and prioritization based on risk levels. These views led participants to favor approaches that prioritize switching the sickest patients last (i.e., those with poorly controlled disease) and that offer patients control and choices around biosimilar switching. Participants also expressed preferences for combining elements of different approaches to maximize fairness.

**Conclusions:**

Approaches to biosimilar switching should consider patients’ desires for transparency and effective communication about biosimilar switching and engagement in their medical decision-making as part of patient-centered care. Incorporating patient preferences around biosimilar switching is critical when navigating the quality and affordability of care in resource constrained settings, both within the VHA and in other healthcare systems.

**Supplementary Information:**

The online version contains supplementary material available at 10.1186/s12913-024-11570-3.

## Background

A patient-centered approach to healthcare emphasizes patient-provider communication, empowerment of patients to actively participate in the medical decision making process, and personalized treatment to meet patients’ specific needs and goals [1, 2]. Patient preferences are a key component of shared decision making and are particularly important in the prescribing of biologics and biosimilar medications. Biologics are among the fastest growing classes of medications that are highly effective in treating many cancers and autoimmune disorders, such as inflammatory bowel diseases (IBD), rheumatoid arthritis, and lupus. However, biologics are among the costliest medications, accounting for 43% of the total prescription drug spending in the U.S. in 2019 [3]. Biologics differ from conventional medications because they are produced in living systems and, therefore, cannot be copied precisely as a “generic” medication. Biosimilars are similar, but not identical, versions of originator biologics that offer significant cost-savings. Consequently, biosimilar switching programs, where patients are switched from an originator biologic to a biosimilar, present an opportunity to mitigate rising drug costs and expand patient access to important biologic therapies [4–6].

In the U.S., biosimilars are not required to go through the same comprehensive clinical trial process as originator biologics but rather a more streamlined 351(k) FDA approval pathway, in which they must demonstrate that they are highly similar and have no clinically meaningful differences in terms of safety and efficacy from the originator. The streamlined approval process has led to safety and efficacy concerns by some patient [7–9], despite accumulating evidence to support the safety and efficacy of biosimilar switching [10, 11]. Studies have also reported decreased patient acceptance and adherence to biosimilar medications, which can lead to loss of treatment response, adverse reactions, and inefficient resource utilization [12, 13]. With more than eighty biosimilar medications in the pipeline and expanding indications for their use [14], it is important to understand patient perceptions and acceptance of biosimilar switching to inform patient-centered care strategies that promote medication adherence and efficient resource utilization.

To date, our understanding of patients’ perceptions of biosimilars and biosimilar switching is based largely on studies using tools such as surveys and focus groups [7–9, 15–18], which have limitations when it comes to informing patient-centered approaches around complex issues like biosimilar switching. Patients may only have a cursory knowledge of these medications [8, 9], thus limiting insights into their preferences around these medications. Democratic deliberation (DD) is a qualitative method that offers a practical and reliable approach to solicit informed and considered opinions on complex issues. DD methods combine education by experts with facilitated peer discussion to provide well-informed lay perspectives and proposals [19–24]. DD methods have proven effective in soliciting the considered opinions of members of the public [25–28], including in healthcare approaches related to prioritization and resource allocation [29–31].

In this study, we used DD methods to understand the perceptions of patients with IBD in the Veterans Health Administration (VHA) on biosimilar medications and biosimilar switching programs. Using IBD as a use case, these findings aim to inform implementation of patient-centered approaches to biosimilar switching in resource-constrained settings, both within and outside the VHA.

## Methods

### Study population

Participants were recruited from two VHA sites, the VHA Ann Arbor Healthcare System and the VHA Houston Healthcare System. Participants were eligible if they: (1) had a diagnosis of IBD, (2) visited a VHA facility in the previous 12 months, and (3) currently or previously received treatment with an anti-tumor necrosis factor biologic medication (originator or biosimilar) as indicated in the VHA electronic health record. In addition, participants needed to have access to a computer, tablet, or smartphone that could support Zoom Video Conferencing (Zoom Video Communications, San Jose, CA). Participants were recruited via mail and telephone. To mitigate technological barriers, we provided each Veteran participant with pre-session tech support and Zoom training. Each of the participants who attended the deliberations received a $200 gift card for their time. Additional information about recruitment methods can be found in Appendix 1. The Institutional Review Boards at the VHA Ann Arbor Healthcare System and VHA Houston Healthcare System approved this study, and participants provided written informed consent before participation.

### Deliberation sessions

#### Overview

We conducted two virtual deliberations in early 2022. VHA patients with IBD took part in 5-hour deliberations over two days via Zoom Video Conferencing. The deliberative sessions included educational presentations, facilitated small group discussions, a larger plenary discussion, and surveys that were completed via mail before and after the deliberation. The complete agenda can be found in Table 1.

**Table 1:** Deliberation agenda

**Day 1**		
**Time**		**Activity & Topic**
12:50–1:00pm		**Logging On** • Please log-in promptly to allow time to fix any glitches!
1:00–1:20pm		**Welcome and Introductions** • Introduction of study team and overview of the day
1:20–2:05pm		**Expert Presentation 1: Background** • Why are we doing this? • What are IBD, Biologics, Biosimilars, and Originators? • What are the benefits/concerns with ‘switching’ medication type?
2:05–2:20pm		**Q & A session with Presenters**
2:20–2:25pm		**Brief Break**
2:25–3:25pm		**Small Group Discussion 1 (Break-out Sessions)** • Brief introductions and icebreaker • Comparing generic vs. biosimilar risk o Participant ranking on: • How *personally* risky is it to switch? • How risky for Veterans *in general*? • What are some reasons Veterans should or should not be informed of the switch to a Biosimilar?
3:25 − 3:30pm		**Day 1 Wrap Up**
**Day 2**		
12:50–1:00pm		**Logging On** • Please log-in promptly to allow time to fix any glitches!
1:00–1:30pm		**Expert Presentation 2: Policies** • What are some of the available policy options regarding: o Which Veterans are switched to Biosimilars. o How Veterans are switched to Biosimilars. • What are some of the factors the VHA is taking into consideration?
1:30–1:40pm		**Q & A session with Presenters**
1:40–2:35pm		**Small Group Discussion 2 (Break-out Sessions)** • Which policy do you prefer and why? • How should these policies be implemented? • Participant ranking of proposed policies • Do you have alternative ideas?
2:35–2:40pm		**Brief Break**
2:40–3:30pm		**Larger Plenary Group Discussion** • Small groups share rankings • Policy discussion • DD Session Feedback

#### Deliberation materials and surveys

Deliberation materials and surveys were developed iteratively by experts in IBD treatment and DD methods and were informed by cognitive interviews with a separate group of patients (*n* = 17) with and without IBD. These interviews included questions about experiences and knowledge of IBD and biologics, comfort level with biosimilars, and perspectives on switching patients with IBD to biosimilars (Cognitive Interview Guide, Appendix 2).

Prior to the DD sessions, participants were mailed a Zoom guide, FAQ sheet, participant guide, presentation slides, baseline survey, and a consent form. After the DD sessions, participants were mailed a follow-up survey. Surveys were used to collect sociodemographic and clinical features as well as to assess IBD and IBD treatment knowledge and attitudes toward the VHA and VHA gastroenterologists (Appendix 3 and 4). The follow-up survey also included questions about biosimilar switching approaches proposed and discussed during the DD sessions.

#### Expert presentations

Two gastroenterologists (AW, JH) and a medical sociologist (RD) gave educational presentations during the 2-day DD sessions. The presentation on Day 1 outlined the purpose of the study, and provided background on IBD, originator biologics and biosimilars, and benefits of and concerns about ‘switching’ to biosimilar medications. The presentation on Day 2 included a review of the main takeaways from Day 1 (including participant input) and an overview of five potential approaches to biosimilar switching, including some initial pros and cons for each approach. Each presentation concluded with a brief question and answer session. The experts were also available to answer questions during the small group discussions.

#### Small group discussions

After expert presentations, each participant was assigned a small group with a trained facilitator. Facilitators were selected based on their experience in facilitation and/or qualitative interviewing. Before the DD session, the facilitators underwent a 2-hour training that included an overview of the session agenda, education on the research topic, and a run through of the small group discussion activities.

On Day 1, participants were asked to rate how risky they believed it was for (1) themselves and (2) for patients *in general* to switch from a name-brand medicine to a generic medication, and how risky it was for (3) themselves and (4) patients with IBD *in general* to switch to a biosimilar (on a 1–10 scale, 1 = Not Risky, 10 = Very Risky). Participants were asked to provide reasons for their responses and to discuss their perspectives on whether and how the VHA should inform patients about biosimilar switching.

On Day 2, participants were asked to individually rank order five hypothetical approaches to biosimilar switching from most (1) to least (5) preferred: (a) “status quo” where switching approaches vary by VHA facility, (b) “sickest last” where patients with the most severe IBD (i.e. less well controlled disease) are switched last, (c) “opt-out” where patients are given a choice to opt-out of switching, (d) “next appointment” where patients are switched at their next appointment, and (e) “lottery” where patients are switched based on random selection. Participants were then asked to provide reasons for their responses, discuss the benefit and risks of different approaches, and come to a consensus as a small group on the most and least preferred approaches for biosimilar switching. Participants were also able to suggest modifications or alternative approaches.

#### Plenary large group discussion

At the end of Day 2, participants came together for a final plenary group discussion to review the overall vote rankings of the different approaches to biosimilar switching. Each group facilitator briefly summarized their small group’s preferences and reasoning, which was then followed by a moderated discussion on participants’ views on the deliberative vote results and any further modifications they would make.

### Qualitative analysis

Small group sessions were recorded, transcribed, and de-identified. The qualitative coding scheme was developed using an interpretative description approach to thematic analysis [32–35]. An initial coding scheme was primarily developed using a subset of the research team (KR, CK and JB) for the small group session questions, as well as facilitator summaries (during large group discussion) and facilitator debriefings (after each deliberation). The coding scheme and qualitative analysis were also reviewed by the entire study team. The coding scheme was then iteratively refined by review of the six transcripts from the Ann Arbor DD by three study team members (KR, CK, JB). Transcripts were then qualitatively coded by two study team members (KR, CK), with differences resolved by consensus discussion. The final four transcripts from the Houston DD were coded by one study team member (KR). Analysis was performed using NVivo qualitative software (QSR International, Doncaster, Australia).

### Statistical analysis

Paired Student’s t-tests were used to compare pre-and post-DD survey responses, as well as responses to questions related to risk of medication switching during the DD sessions. Data were presented as mean±SD, and a p-value of < 0.05 was considered statistically significant. Given the small sample size, we did not evaluate the remaining quantitative data for statistically significant associations. This manuscript primarily focuses on a qualitative analysis of the small group discussions to identify important themes.

## Results

Invitations were sent out to 175 Veterans. Thirty-one participants provided written consent to participate, and 29 (*n* = 17 in Ann Arbor and *n* = 11 in Houston) attended and completed the virtual deliberation sessions. We convened a total of 5 facilitated small groups (3 in Ann Arbor and 2 in Houston) with 5–6 participants per small group. Participants were predominantly male (66%) and white (69%), with a mean age of 59 years. Sociodemographic and clinical features can be found in Table 2.

**Table 2:** Participant characteristics (*n* = 29)^a^

		Total *N* (%)
**Gender**		
	Female	10 (34)
	Male	19 (66)
**Age**,** Mean** ± **SD**		59 ± 12.2
**Race/Ethnicity**		
	White	20 (69)
	Black	6 (21)
	Other	3 (10)
**Education**		
	High School Diploma/GED or less	2 (7)
	Some College or 4-year degree	22 (79)
	More than 4-year college degree	4 (14)
**Annual household income**		
	$39,999 or lower	10 (36)
	$40,000 or higher	13 (46)
	Prefer not to answer	5 (18)
**Employment**		
	Working full time or part time	11 (38)
	Unemployed, retired or disabled	18 (62)
**IBD Type**		
	Ulcerative Colitis	11 (38)
	Crohn’s Disease	18 (62)
**Disease Symptom Severity (In the last 6 months)**		
	Constant/Often	5 (17)
	Sometimes/Occasional	14 (48)
	Rare/None	10 (35)
**Current Biologic Medication Type**,** if any IBD Medication**		
	Originator (e.g., Adalimumab, Certolizumab)	15 (52)
	Biosimilar (e.g., Infliximab)	8 (28)

^a^ Valid percentages of non-missing data are shown

**Fig. 1:**
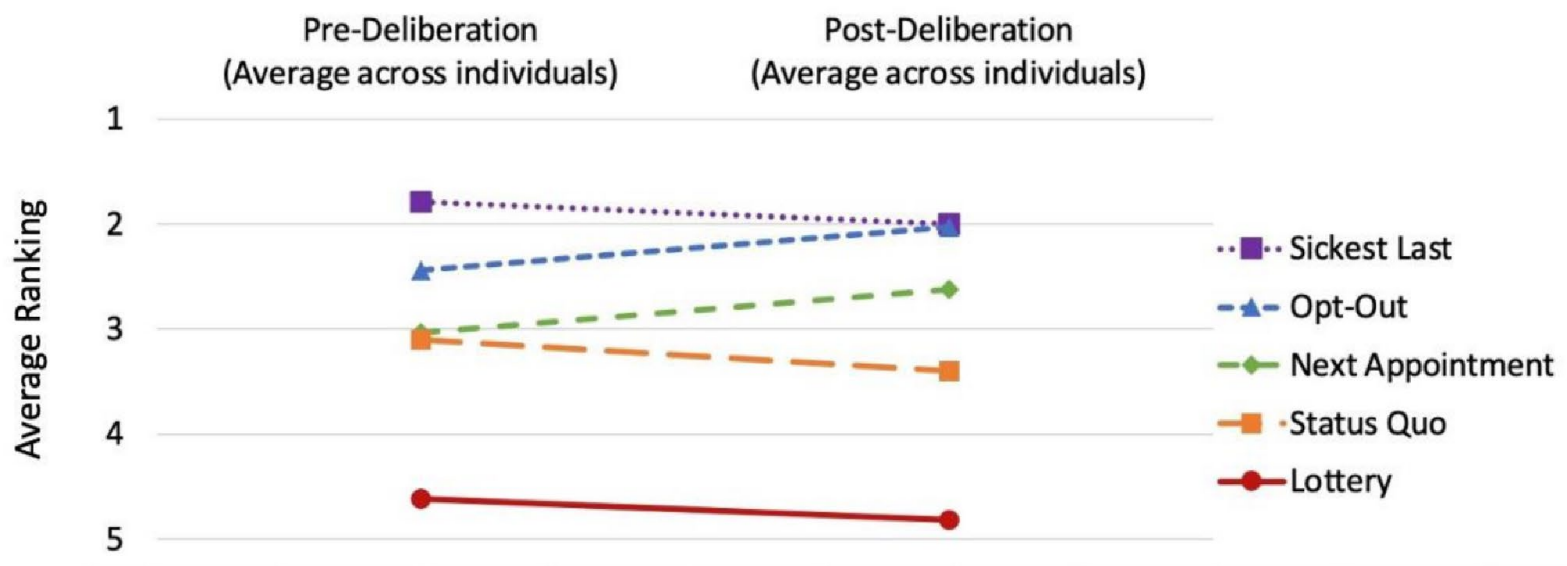
Rankings of hypothetical biosimilar switching approaches. At the start of the small group discussion on Day 2, participants (*n* = 29) were asked to individually rank order five hypothetical approaches for biosimilar switching from most (1) to least (5) preferred: (1) “status quo” where switching policies vary by VHA facility, (2) “sickest last” where patients with the most severe IBD are switched last, (3) “opt-out” where patients are given the choice to opt-out of switching, (4) “next appointment” where patients are switched at their next appointment, and (5) “lottery” where patients are switched based on random selection. Participants were asked to rank the approaches again in the post-deliberation survey via mail

Participants rated switching to a biosimilar medication as significantly riskier than switching to generic medications for themselves and for patients in general (Table 3). Prior to the small group discussions, most participants preferred the “sickest last” approach (1.8±0.9, on a 1–5 scale, 1=“most preferred” and 5=“least preferred”) followed by “opt-out” (2.4±1.4), “next appointment” (3.0±1.3), “status quo” (3.1±1.0), and “lottery” (4.6±0.9). These rankings did not significantly change after the deliberation session (Fig. 1).

**Table 3:** Perceived risk of medication switching (*n* = 28)^a^

How risky you believe it is for…		Mean^b^ (SD)	*P* value^c^
1.	…you *personally* to switch from a Brand med to a *generic*?	3.39 (2.43)	*p* < 0.001
2.	…you *personally* as a patient with IBD to switch to a *Biosimilar*?	5.03 (2.55)	
3.	…Veterans *in general* to switch from a *Brand med* to a generic?	3.71 (2.14)	*p* < 0.001
4.	…Veterans with IBD *in general* to switch to a *Biosimilar*?	5.02 (1.89)	

^a^ One participant was unable to complete the ranking due to technical difficulties.

^b^ Responses on a 1–10 scale, 1 = Not Risky, 10 = Very Risky

^c^ Paired t-tests were used to compare mean response to question 1 vs. 2 and question 3 vs. 4.

In response to 6 true/false IBD knowledge questions, participants showed significantly increased knowledge of IBD (3.52±1.55 to 5.03±0.61, *p* < 0.001) at post-survey. Participants felt their opinions were respected by their small group (9.1±0.99, 1 = Not at all to 10 = Very Much), they were listened to by their facilitator (9.4±0.78), that the small group process was fair (9.5±0.74), and participants agreed that they were willing to abide by the preferred approach by their small group, even if it was not their preference (8.2±0.1.63). Participants also indicated they trusted the VHA (3.45±0.77, 1 = Not true, 4 = Very true) and VHA health care providers (3.48±0.72).

### Perceptions of Biosimilar switching

Five major themes emerged from the small group discussions around (1) perceived risk of biosimilar switching, (2) desire for transparency and information, (3) shared decision making, (4) considerations of cost and access, and (5) personalization and prioritization in health care. Additional exemplary quotes are presented in Appendix 5.

#### Theme 1: Perceived risk of biosimilar switching

Participants across all small groups expressed concern about the potential risks associated with biosimilar switching. Participant concerns included potential adverse consequences from being switched, such as reduced efficacy, symptom re-emergence, and development of an immune response that would prevent switching back to the originator.

*I have a great concern that a biosimilar would not work and those symptoms would start creeping in and when they’ve indicated even more so that it’s possible that going back to the original biologic drug would not be effective anymore and that they would have to start trying the other biologics that are out there*,* that creates a very big*,* big concern for me.*

One participant pointed out that with generic medications individuals were simply paying (more) for the brand name, but with biosimilars, the balance between risks and benefits were less clear.

*[Generics] all have the same ingredients*,* what are you doing? You’re paying for the name. As far as the biosimilars and the biologics I’m not really sure because I’m not very educated on them. My biggest fear is*,* I know everything has a risk*,* you take a risk by leaving your house every day. Everything you do in life has a risk. What are the benefits? We need to figure out what are the benefits of going from a biologic to a biosimilar? Medically standing is it going to improve a person’s quality of life? If it does*,* then obviously the benefits are going to outweigh the risk.*

#### Theme 2: Transparency and information

Participants emphasized the importance of transparency in healthcare and keeping patients informed in general. Participants also discussed the importance of communication with patients about changes in their medications, including information about risks and benefits: “*Everybody should be informed on everything they’re getting. You need to know why you’re getting it*,* what is it doing for you*,* what is it*,* what are you going to expect*,* [and] is it going to make any of your symptoms worse?”* They felt that transparency was especially important in the case of IBD and biosimilars because of their uncertainty about the possible health consequences of minor variations between originators and biosimilars. A few participants pointed out that transparency was an ethical requirement in the context of risk and that keeping patients in the dark would diminish trust in the healthcare system.

*I don’t think it’s ethical to change someone without telling them that you’re going to change them because there is the remote risk of whatever you know*,* with those so if you do other changes you have to tell the patients*,* right? Or they have to tell us. It’s not ethical to not tell.*

One participant felt the ethical need to inform patients trumped potential cost-savings of switching patients to biosimilars: “*I mean somebody is already on medicine for Crohn’s or whatever*,* you definitely would have to inform them*,* right? I would want to know. And if that causes fewer resources*,* you’ll have to figure that one out.”* Several participants pointed out that they would want to be informed about biosimilar switching so they could better manage their care – by keeping track of their medications and monitoring their symptoms for any changes due to medication. However, one participant did note that there was a potential risk of informing patients if they did not fully understand the reasoning behind the switch.

*If somebody is informed but doesn’t have the proper understanding […] they would freak out because they would think that it was a quality issue that they were being substituted for rather than you know*,* a monetary thing or something of that nature.*

#### Theme 3: Shared decision making

Transparency and information about biosimilar switching were necessary but insufficient for most of our participants. Participants wanted patients to have a role in decision making when discussing treatment switches with a doctor, pharmacist, or other trusted healthcare provider. Some participants discussed an informed consent model of decision making. For example, when one participant insisted that the doctor should be the one to decide, another responded that it was essential to have a patient’s informed consent.

*1st Participant: The doctor should make the decision I think about opting out. They have so much more information than we do. 2nd Participant: I’d agree with that*,* but I’d have to say with the Veteran’s consent too*,* not him not knowing*,* you know what I’m saying?*

Participants emphasized the importance of provider-patient communication around biosimilars. Participants expressed trust in their providers to convey accurate information and to know and do what is best for their patients. At the same time, some participants pointed out that both the provider and patient have important roles in deciding whether to switch to a biosimilar.

*… [doctors] are the professionals*,* we trust in what they do and we trust that they have the skill set to tell us what’s necessary for our own treatment*,* but at the same time I think that we do have some responsibility to understand what’s going into our bodies and so with that being said I think it’s absolutely vital to have that conversation…*.

One participant pointed out that discussing the switch with one’s provider might make it less scary, and therefore, individuals may be less likely to opt out.

*… if they had the doctor talking to the patient*,* giving him the chance yes*,* maybe to opt out but more persuasive in the sense that it may not be needed. See it’s the scary part in there again. Well*,* am I going to get sicker? So*,* if the doctor’s talking to them*,* they might not get so afraid.*

#### Theme 4: Cost and access

Participants had diverse attitudes toward how to balance the need for transparency and shared decision making with other health system priorities including reducing costs and increasing access to care. A few participants supported biosimilar switching because of its cost savings and potential to increase access.

*We’re talking about something that could be beneficial to every Veteran that has some sort of disease that requires some product and that maybe a Biosimilar’s made for and if they can do a Biosimilar for a less price then it’s going to help every Veteran out.*

Some participants recognized that biosimilars would reduce costs of care but also emphasized the importance of discussing switches with the provider to promote transparency and awareness. Other participants felt that switching was not worth the risk and expressed discomfort with treatment approaches focused on saving money: “*It shouldn’t be forced on you to go a cheaper route to save money because in the end you’re risking your life*,* you’re the one that’s got to pay that cost if something bad happens*,* you’re the one that’s going to pay for it…”*.

#### Theme 5: Personalization and prioritization

Participants across all small groups advocated for a personalized approach to care based on a patient’s needs. Some participants noted that it was essential to consider that people’s bodies are different, and they may have different responses or reactions to medication. One participant emphasized the importance of coordinating with healthcare providers to monitor and adjust for these individual differences.

*… everybody is going to react differently at different times. Something might work fine for you for one year and then next year*,* it doesn’t work at all so you have to have that inner connection with the system*,* with a doctor or a practitioner of some sort so that they can adjust things…*.

Recognizing that patients have different circumstances, needs, and risk levels, participants in our small groups were generally supportive of policies that prioritized switching patients based on some criteria. For example, some participants felt that new patients should be put on biosimilars first (with close follow-up). Alternatively, one participant felt that patients should be prioritized by risk, with those with lower risk (i.e., patients with well-controlled disease) being switched first. The data about these more straightforward cases could then be used to inform the next steps.

### Participant preferences towards Biosimilar switching approaches

Overall, participants supported “sickest last” and “opt-out” approaches over “next appointment,” “status quo,” and “lottery” approaches to biosimilar switching.

#### Preferred approaches: “sickest last” and “opt-out”

Participants’ views on the importance of personalization, prioritization, and risk reduction for the most vulnerable patients led participants to rank “sickest last” as their most preferred approach to biosimilar switching.

*I think the sickest being put last is the best solution because like I said*,* it’s part of your life*,* the Veteran. Yeah*,* it’s probably going to switch us all eventually anyway but the sickest last*,* at least see how it works in other Veterans and if it works well before you switch if the Veteran is really sick.*

Others had reservations about the “sickest last” approach because it did not include patient control and consent. Many participants also valued patient control and choice regarding biosimilar switching, which led to a preference for an “opt-out” approach: “*I definitely think it’s important you know*,* the opt out even though our doctor knows the best care for us*,* we still need an opinion as well. Our voice needs to be heard whether we want to take it or not…”* Participants had concerns about the risks of switching and the lack of data about the effects of changing medications for IBD patients. However, a few stressed the need to make an *informed* decision about opting out and express concern about the possible cost to the VHA of giving patients a choice about switching: “*…maybe the Vet is just choosing the most expensive drug out there because he doesn’t want to have a switch or doesn’t want to change at all*,* doesn’t want anything to do with these Biosimilars and that’s not really good for the VA…”*.

#### Less preferred approaches

Participants had mixed views of the “next appointment” approach. Participants liked that the next appointment would provide the opportunity for patients to discuss biosimilar switching with their providers and be more fully informed: “*I picked as my number one next appointment because that’s really when you can get all of your questions answered by your physician…”* However, others were concerned that patients would be switched automatically and not be part of the decision making: “*… if you’re saying that you couldn’t say no at the next appointment then I would not put that as number one.”* The “status quo” approach was less often discussed in the small groups, but participants did express reservations about an approach in which switching varied by institution and physicians: “*I would just prefer it to be a common*,* coherent and coordinated policy*,* not kind of disjointed across various areas.”* Participants ranked the “lottery” approach as the least preferred of all options because it did not make room for personalized care and prioritization. Participants highlighted the potential unfairness of an approach that did not consider these factors: “*I think treatment medication should be gauged towards the patient not just if my number comes up or not.”*

The challenge of finding one implementation approach that is fair and works for everyone was mentioned by several participants. Although the deliberation was designed to elicit opinions about the risks and benefits of each individual approach, participants were inclined to suggest ways of combining components of several approaches to maximize fairness.

*No two people are the same so it’s always going to be kind of tricky to try to pinpoint policy and make it work for all. So*,* my thing would be to try to add on as many policies as I could so the more policies that could be adopted*,* I think the more fair it would be for as many people as it could.*

## Discussion

The primary goal of this qualitative study was to use DD methods to elicit the informed and considered opinions of patients with IBD on biosimilar switching in the VHA. Our findings indicate that patients with IBD do not perceive biosimilars as the equivalent of a “generic” medication. Participants perceived switching from an originator biologic to a biosimilar as significantly riskier than switching from a traditional brand name medication to a generic medication. Five major themes also emerged from our deliberative sessions with VHA patients with IBD: (1) concerns about the risks and benefits; and the importance of (2) patient-provider communication and transparency; and (3) shared decision making and patient involvement in treatment decisions; as well as (4) considerations when balancing cost-saving with other priorities; and (5) the importance of individualized care and prioritization based on risk levels. These views led participants to favor approaches that prioritize switching the sickest patients (less well-controlled disease) last and that offer patients control and choices around biosimilar switching, but they also expressed preferences for combining elements of different approaches to maximize fairness. These findings support patient engagement in the development of biosimilar switch programs as part of patient-centered care.

Our study confirms the conclusions of research on patient attitudes about switching to a biosimilar. A small focus group study done with patients and caregivers in the UK, found that like our participants, there was a willingness to switch to a biosimilar if it would save money for the health system – in their case the National Health System – and if that money would be used to treat others [36]. The results of another UK study, involving interviews with health care professionals, were similar. In addition to concerns about safety and efficacy, they noted the need to address the opinions of patients and to be transparent about how cost savings would be shared [37]. A survey study of patients with rheumatologic diseases done in the US found general satisfaction with the switch to biosimilars, but emphasized the need to involve patients in the switching decision-making process to allay concerns and enhance uptake of the biosimilar [38].

Our work, relying democratic deliberation, extends this research by educating, engaging, and dialoging with patients. Given the complexities involved in switching from originator biologics to biosimilars, it is necessary to go beyond surveys and interviews to challenge and probe participant opinions and the values that shape those opinions. DD offers a practical and reliable way of doing that. There are, however, limitations to this study. Like all studies of human behavior, selection bias cannot be ruled out. Although we had reasonable representation of race/ethnicity and gender (see Table 1), biases may have been introduced by things like, for example, level of comfort with the technology required for online meetings and degree of concern about health. Carrying out deliberations via Zoom may have served as a barrier to participation for some Veterans. To overcome technological barriers, we provided pre-session tech support and Zoom training for each Veteran participant and mailed them instructions and tips for using Zoom. We also trained facilitators on how to foster engagement in the Zoom breakout room setting. However, virtual deliberations also allowed Veterans with IBD to participate from locations most convenient to them, reducing the burden of attendance and expanding access to individuals who may have been excluded from in-person deliberation [39]. Additionally, the sampling method employed may have introduced a selection bias towards individuals more willing to volunteer or those holding stronger opinions, potentially limiting the diversity of viewpoints. The generalizability of these deliberative results may also be limited, as they reflect the perceptions and experiences of predominantly white, male veteran Veterans with IBD from two VHA clinic sites. Future studies should include patients managed outside the VHA or those with non-IBD autoimmune disorders. Finally, we used an assessment of risks scale that was not validated, and while we did note significant differences in perceived risks of biosimilars and generic drugs, the primary purpose of the tool was to solicit participants’ reasoning related to their risk ratings.

Nonetheless, these findings help us to develop a better understanding of patients’ informed views of biosimilar switch programs and has implications for future practice. The study highlights that patients with IBD do not perceive biosimilars as equivalent to originators, indicating the need for targeted educational efforts to address these concerns. Healthcare systems considering biosimilar switching programs should prioritize providing patients with comprehensive information to facilitate informed decisions in collaboration with their healthcare providers. This study underscores the importance of patient engagement and sharing accurate information to support successful implementation of biosimilar switch programs. By doing so, healthcare providers can improve patient satisfaction, optimize treatment outcomes, and utilize resources more efficiently.

## Conclusion

There is a growing body of evidence showing the value of medical decision-making that involves both patients and providers. Not only are patients more satisfied with their care, they are more likely to comply with treatment, and outcomes are better [40–43]. Our study adds a new dimension to the process of shared decision making by showing the importance of including the insights and values of the community when creating health policies that affect that community. In our case, we learned that in the resource-limited setting of the VHA, patients with IBD favor biosimilar switching approaches that prioritize switching the sickest patients last and that offer patients control and choices around switching. What we learned, and how we learned it, will be especially valuable as health systems face the difficult task of providing patient-centered care without sacrificing quality and affordability. Soliciting and responding to patient preferences promotes ownership over the policy process that will help those who manage health systems to navigate the challenges they face.

## Electronic supplementary material

Below is the link to the electronic supplementary material.

Supplementary Material 1

Supplementary Material 2

Supplementary Material 3

Supplementary Material 4

Supplementary Material 5

## Data Availability

The full dataset is not publicly available due to confidentiality policies. Some additional data may be made available upon reasonable request.

## Abbreviations

IBD: Inflammatory Bowel Disease
VHA: Veterans Health Association
DD: Deliberative Democracy
