# Supplementary material for: Public deliberation to assess patient views on biosimilar medication switching for the treatment of inflammatory bowel disease
Source: BMC Health Serv Res. 2024 Oct 9;24:1209. doi: 10.1186/s12913-024-11570-3 (PMC11462922; doi:10.1186/s12913-024-11570-3)
Supplement: Supplementary file 1 — Supplementary Material 1 [file 12913_2024_11570_MOESM1_ESM.docx]

**Supplemental Recruitment Methods**

Eligible participants were 1) Veterans diagnosed with IBD who were either currently or previously on infusion anti-TNF’s; 2) Veterans who had a diagnosis of Diabetes and were currently on a biosimilar medication; 3) Veterans who were diagnosed with IBD and Diabetes; 4) At least one visit to a VA facility in the past 12 months; 5) Patients without dementia or other significant mental impairment noted in their medical record. An attempt was made to stratify participants by location, gender, race, and diagnosis using a sampling matrix developed by both clinical and qualitative experts.

The data manager for this project pulled batches of 10 potential participant names at a time and recruitment was done on a rolling basis until the recruitment window passed and an adequate number of participants was achieved. Participant information was included in a study database which was stored behind the VA firewall on a VA research study folder on VA servers.

175 eligible participants were mailed invitations, thirty-one participants provided written consent to participate, and 29 (n=17 in Ann Arbor and n=11 in Houston) attended and completed the virtual deliberation sessions.
