# Supplementary material for: Public deliberation to assess patient views on biosimilar medication switching for the treatment of inflammatory bowel disease
Source: BMC Health Serv Res. 2024 Oct 9;24:1209. doi: 10.1186/s12913-024-11570-3 (PMC11462922; doi:10.1186/s12913-024-11570-3)
Supplement: Supplementary file 2 — Supplementary Material 2 [file 12913_2024_11570_MOESM2_ESM.docx]

**Appendix 2. Cognitive Interview Guide**

**Methods**

Specific content and questions for the deliberative session were developed in part by conducting individual interviews with patients from the Ann Arbor and Houston VHA Medical Centers. Inclusion criteria included patients: 1) diagnosed with IBD who are either currently or previously on infusion anti-TNFs; 2) who are diagnosed with diabetes and are currently on biosimilar medication; 3) diagnosed with both IBD and diabetes; and 4) At least 1 visit to a VA facility in past 12 months. Patients with dementia or other significant mental impairment noted in their medical record were excluded. Diabetic patients were included to give us a broader range of experiences with biosimilars.

Recruitment was stratified by gender, race, and diagnosis.

- A total of 17 patients were interviewed (9 Male, 8 Female).
- 8 patients with Ulcerative Colitis, 14 patients with Crohn’s Disease, 4 patients with Diabetes only
- 14 patients on Biologic medications; 3 patients on biosimilar medications

**Semi-structured Interview Guide:**

**Patient Preferences on Use of Biosimilar Medications**

**Introduction**

Hello, Mr./Ms. _____________________, this is ___________. I am calling on behalf of the VHA for a research project you recently learned about from one of my colleagues. [I/they] spoke to you [at your clinic appointment/on the phone] about doing an interview for a project called, **“Patient Preferences on Use of Biosimilar Medications”**. This interview should take approximately **30-45 minutes**.

**Is it still a good time to talk?**

*[If yes, continue; If no, reschedule. Up to 2 reschedule dates are allowed.]*

Before we begin, I would like to take a minute to remind you why we are asking Veterans like you to participate in an interview. This interview will help us develop information and materials to improve the way we discuss potential policies for treating Veterans with Inflammatory Bowel Disease (which includes Crohn’s disease or ulcerative colitis).

Participation in this interview is completely voluntary. If at any time or for any reason you would prefer not to answer any questions, please feel free not to answer. If at any time you would like to stop participating, please tell me. You will not be penalized in any way for deciding to stop participation at any time. What you say will not affect your care at the VHA.

I will be taking notes during the interview, but I would also like to record this interview to make sure that I remember accurately all the information you provide.

Any information you provide will be handled in a confidential manner. Only people working on this study will use the interview notes and recordings. We will take steps to ensure your answers stay confidential.

1. Transcribed interview – if we choose to get the recordings transcribed, your name will not appear on any of the transcripts. Each audio file will be labeled only with a study ID number. The file will be kept on a secure server.
2. Audio – this will be saved on our secure server and deleted off the recording device.
3. Interview notes – these will be typed up and saved in a folder separate from transcribed interviews

You will be given a **$25.00 [*MEIJER/WALMART*] gift card** as a small token of our appreciation for your assistance with our research project.

**Do you have questions before we begin?**

**Are you still interested in participating in this interview?**

*[If no longer interested, say:]*

That is no problem at all, thank you very much for your time. Good-bye.

*[If the participant agrees to participate, say:]*

**Is it okay if I turn on the recorder now?**

*[Turn DVR on and state the following:]*

This is ________________ [*name of interviewer*].

Today’s date is ____________

Time is _____________

Participant ID is ____________

**General Questions**

I have a few general questions to begin:

- **In what branch of the military did you serve?**
- **How long did you serve?**
- **What is the name of your VHA primary care doctor?**

**Patient IBD Knowledge**

For this next set of questions, we just want to get a sense of what people know about Inflammatory Bowel Disease. There are no right or wrong answers.

1. **Have you ever been diagnosed with, or told that you have, Inflammatory Bowel Disease (such as Crohn’s disease or ulcerative colitis)?** *[If “No”, Skip to Question 2]*
   1. **Specifically, were you told you have Ulcerative Colitis, Crohn’s Disease, or something else?**
   2. **Who told you?**
   3. **How did you feel when you were told this?**
   4. **What did [*the doctor/nurse/health care provider*] tell you about Inflammatory Bowel Disease?**
   5. **Can you tell me what else you know about Inflammatory Bowel Disease, in general?**
   6. **Have you ever received treatment for Inflammatory Bowel Disease?**
      - *[If pt has had treatment]*
        1. **Would you mind telling me about your treatment experience?**
        2. **What do you know about the medicines that you have used?**
      - *[If no treatment]*
        1. **Would you mind telling me why you have not received treatment?**
2. **Tell me what you know, in general, about treatment for Inflammatory Bowel Disease.**

**Probes**:

- Tell me your understanding of how effective treatment is.
- Tell me your understanding of how well treatment is tolerated (i.e. side effects).
- Tell me your understanding of how much it costs to treat Inflammatory Bowel Disease.

1. **Have you ever heard of drugs called “biologics”?**
   1. **What do you know about biologics?**
2. **Have you ever heard of the terms “originator” or “biosimilar”?**
   1. **What do you know about originators and/or biosimilars?**

**Education**

Now I’d like to tell you more about treatments for Inflammatory Bowel Disease. You may already know about these treatments, but we want to make sure everyone we interview has the same information. Please note that **we are not medical professionals and cannot offer medical advice**; please speak to your doctor if you have medical questions or concerns about treating IBD.

There are **different *types* of medicines** that can treat most people with Inflammatory Bowel Disease as well as other diseases, such as Diabetes.

One type of medicines is called **biologics, which** are made from natural proteins. These medicines are often given by IV infusion or injection. One example of a biologic used to treat Inflammatory Bowel Disease is Remicade. Insulin, used to treat Diabetes, is another common example of a type of biologic.

Biologics are called either **originators** or **biosimilars**. **Originators** are the original version of the medication. **Bio-similars**, on the other hand, are designed to be very similar to the original version. For example, Inflectra and Renflexis are biosimilars for the originator Remicade. There are also biosimilars for various kinds of insulin.

Note that biosimilars are ***not* the same as a generic version** of a brand medication. *Generics* are essentially chemically identical to the original drug; however, *biosimilars* are chemically different from their originators.

It is also important to note that the **FDA has approved** the use of biosimilars, but the agency has not weighed in on whether they are as effective as or have the same number of side-effects as the originators.

**I know that was a lot of information, can I repeat any of that for you?**

1. **What is your experience, if any, with originators or biosimilars—either in the context of IBD or Diabetes?**

**Probe:** Have you ever heard of Remicade, Inflectra, Renflexis, or some other biologic?

1. **Given what we have discussed so far, do you think you would you be More, Less, or Equally comfortable receiving a Biosimilar for treatment of IBD, as compared to an Originator? (And Why?)**

**Policy**

**The VHA needs to create a policy for the use of biosimilars, and we are asking Veterans for advice. There are a couple of issues here.**

- One of the main advantages of biosimilars is that they are **significantly less expensive** than originators. So, switching patients to biosimilars can save the VHA on limited resources.
- However, because biosimilars and originators are not *identical* there may be some differences in side-effects or outcomes. So, it seems reasonable that **patients may want to be informed about the switch and/or give their approval** before being switched to a biosimilar. This would:
  - Take additional time,
  - May require an additional appointment, and
  - May result in fewer patients being switched to the less expensive biosimilars.

All of these things will result in a **greater drain on limited VHA resources**.

1. **Given what we have just discussed, do you think the VHA should switch a substantial number of IBD patients being treated with Originators over to Biosimilars? (And Why?)**
   1. **[*If No (probe)*]:** Some are concerned that originators are very costly, so if a lot of patients opt for originators only, this would drain limited VHA resources. How would you respond to those concerns?
   2. **[*If Yes (probe)*]:** Some are concerned that biosimilars are not identical to originators and that some patients have reported they prefer the originators. How would you respond to those concerns?
2. **Who should make decisions about switching patients to biosimilars?**

**Probe:** Should Physicians, Patients, The VHA System, or some other entity should make these decisions?

**Probe:** What is a patient’s role in making decisions about switching?

1. **Should patients be informed that they are being switched from an Originator to a Biosimilar? (And Why?)**
2. **How would *YOU* feel if your doctor switched your medication from an Originator to a Biosimilar without your knowledge? (And Why?)**
3. **If the VHA *does* inform patients about switching their medication from an Originator to a Biosimilar, *how* should patients be informed?**

**Probes:** Should doctors first meet with their patients before changing their medication?

**Probes:** Should patients be informed via email, a physical mailing, a phone call, or some other method?

1. **How much control should patients have over whether they are switched to a Biosimilar?**

**Probes:** Should patient approval be required before switching them?

**Probes:** Should patients be able to refuse the change in medication?

1. **IF patients *do* need to give their approval, *when* should this happen: Should they give blanket approval when their treatment starts, or should they give approval each time their medication is changed?**
2. **If you were in charge, how would you balance the need of the VA to provide the best care for patients with IBD while still ensuring they conserve sufficient resources to treat Veterans with other conditions?**

**Probes:** What do you see as the benefits/harms of your approach/policy?

**Probes:** How do you think your approach/policy would work? [Or how could we make it work?]

**Those are all the questions I have; Is there is anything else you would like to add?**

**Logistics**

Thank you very much for your time. I will be mailing you a $25 gift card in the mail.

**Glossary**

**Inflammatory Bowel Disease** is a general term for diseases that cause long-lasting inflammation of the digestive tract. **Both** Ulcerative Colitis and Crohn's disease usually involve severe diarrhea, abdominal pain, fatigue, and weight loss. **Inflammatory Bowel Disease is *not* the same as irritable bowel syndrome**. Inflammatory bowel disease is far more serious and debilitating.

**Ulcerative Colitis** causes long-lasting inflammation and sores (or ulcers) in the colon and rectum.

**Crohn’s disease** causes inflammation deep in the tissues of the digestive tract.
