# Supplementary material for: Public deliberation to assess patient views on biosimilar medication switching for the treatment of inflammatory bowel disease
Source: BMC Health Serv Res. 2024 Oct 9;24:1209. doi: 10.1186/s12913-024-11570-3 (PMC11462922; doi:10.1186/s12913-024-11570-3)
Supplement: Supplementary file 3 — Supplementary Material 3 [file 12913_2024_11570_MOESM3_ESM.docx]

Patient Preferences on Use of Biosimilar Medications

Survey 1

**Survey Instructions**

This survey asks your opinions about treating Veterans with Inflammatory Bowel Disease. We may use this information to make recommendations to the Department of Veterans Affairs on future decisions when treating Veterans with Inflammatory Bowel Disease.

Please answer each question to the best of your ability. You may also choose not to answer any question.

| **Knowledge of Inflammatory Bowel Disease and Treatment** |
| --- |

The **purpose** of these questions is to find out what you currently know about Crohn’s and Ulcerative Colitis (Inflammatory Bowel Disease) and its treatment. You may not know the answers and that is okay.

1. Select one answer from each row.

|  | True | False | Don’t Know |
| --- | --- | --- | --- |
| 1. There is no cure for Inflammatory Bowel Disease |  |  |  |
| 1. Irritable Bowel Syndrome is the same condition as Inflammatory Bowel Disease. |  |  |  |
| 1. Inflammatory Bowel Disease is an unpredictable disease, with periods of flares and remission. |  |  |  |
| 1. In both Ulcerative Colitis (UC) and Crohn’s Disease, the entire digestive tract can become inflamed. |  |  |  |
| 1. People can stop responding to Inflammatory Bowel Disease treatment over time. |  |  |  |
| 1. Inflammatory Bowel Disease is caused by a prolonged state of inflammation that causes injury to the gut. |  |  |  |
| 1. A Biosimilar is a type of medication made from proteins that can be used to help treat Inflammatory Bowel Disease. |  |  |  |
| 1. A Biosimilar is the original version of a biologic medication. |  |  |  |
| 1. The Food and Drug Administration (FDA) has approved the use of Biosimilar medications. |  |  |  |
| 1. A Biosimilar for Inflammatory Bowel Disease is the same thing as a generic version of a brand medication. |  |  |  |
| 1. Biosimilar medications are much less expensive than Originators. |  |  |  |
| 1. The VA has not yet started treating Inflammatory Bowel Disease patients with Biosimilars. |  |  |  |

| **Your Views on the VA** |
| --- |

1. Where do you receive the majority of your health care?

VA

Non-VA

1. How satisfied are you with the care you receive at your VA facility?

Very satisfied

Satisfied

Neither

Dissatisfied

Very dissatisfied

[See next page]

1. The next questions are about your opinion of the **VA Healthcare system**, in general. For each statement below, please check how strongly you agree or disagree.

|  | Strongly Disagree | Disagree | Neutral | Agree | Strongly Agree |
| --- | --- | --- | --- | --- | --- |
| - 1. The VA Healthcare System does its best to make patients’ health better. |  |  |  |  |  |
| - 1. The VA Healthcare System covers up its mistakes. |  |  |  |  |  |
| - 1. Patients receive high quality medical care from the VA Healthcare System. |  |  |  |  |  |
| - 1. The VA Healthcare System makes too many mistakes. |  |  |  |  |  |
| - 1. The VA Healthcare System puts saving money above patients’ needs. |  |  |  |  |  |
| - 1. The VA Healthcare System gives excellent medical care. |  |  |  |  |  |
| - 1. Patients get the same medical treatment from the VA Healthcare System, no matter what the patient’s race or ethnicity. |  |  |  |  |  |
| - 1. The VA Healthcare System lies to make money. |  |  |  |  |  |
| - 1. The VA Healthcare System experiments on patients without them knowing. |  |  |  |  |  |

1. The next questions are about your opinion of your **VA Gastroenterologist (physicians specializing in diseases of digestive tract)**. For each statement below, please check how strongly you agree or disagree

|  | Strongly Disagree | Disagree | Neutral | Agree | Strongly Agree |
| --- | --- | --- | --- | --- | --- |
| - 1. I doubt that my VA Gastroenterologist really cares about me as a person. |  |  |  |  |  |
| - 1. My VA Gastroenterology care team is usually considerate of my needs and puts them first. |  |  |  |  |  |
| - 1. I trust my VA Gastroenterology care team so much that I always try to follow their advice. |  |  |  |  |  |
| - 1. If my VA Gastroenterology care team tells me something is so, then it must be true. |  |  |  |  |  |
| - 1. I sometimes distrust my VA Gastroenterology care team’s opinion and would like a second one. |  |  |  |  |  |
| - 1. I trust my VA Gastroenterology care team’s judgement about my medical care. |  |  |  |  |  |
| - 1. I feel my VA Gastroenterology care team does not do everything they should for my medical care. |  |  |  |  |  |
| - 1. I trust my VA Gastroenterology care team to put my medical needs above all other considerations when treating my medical problems. |  |  |  |  |  |
| - 1. My VA Gastroenterology care team are experts in taking care of medical problems like mine. |  |  |  |  |  |
| - 1. I trust my VA Gastroenterology primary care team to tell me if a mistake was made with my treatment. |  |  |  |  |  |
| - 1. I sometimes worry that my VA Gastroenterology care team may not keep the information we discuss totally private. |  |  |  |  |  |

1. For the statement below, please check how strongly you agree or disagree.

The next questions are about your opinion of your **VA Healthcare system,** in general. For each statement below, please check how strongly you agree or disagree.

|  | Strongly Disagree | Disagree | Neutral | Agree | Strongly Agree |
| --- | --- | --- | --- | --- | --- |
| - 1. I trust the *VA Healthcare System* to allocate limited resources appropriately. |  |  |  |  |  |
| - 1. I trust the *VA Healthcare System* to respect the treatment preferences of Veterans. |  |  |  |  |  |
| - 1. I trust the *VA Healthcare System* to reduce unnecessary healthcare expenses. |  |  |  |  |  |
| - 1. I trust the *VA Healthcare System* to decide which IBD medications to make available to Veterans. |  |  |  |  |  |

The next questions are about your opinion of your **VA health care providers** (primary care doctor, gastroenterologists, etc…). For each statement below, please check how strongly you agree or disagree.

|  | Strongly Disagree | Disagree | Neutral | Agree | Strongly Agree |
| --- | --- | --- | --- | --- | --- |
| 1. I trust my VA *health care providers* to allocate limited resources appropriately. |  |  |  |  |  |
| 1. I trust the *VA health care providers* to respect my treatment preferences. |  |  |  |  |  |
| 1. I trust the *VA health care providers* to reduce unnecessary healthcare expenses. |  |  |  |  |  |
| 1. I trust the *VA health care providers* to decide which IBD medications to prescribe to Veterans. |  |  |  |  |  |

1. All things considered, the VA Healthcare System can be trusted

| Not true | Somewhat true | Fairly true | Very true |
| --- | --- | --- | --- |
| 1 | 2 | 3 | 4 |

1. All things considered, VA health care providers can be trusted

| Not true | Somewhat true | Fairly true | Very true |
| --- | --- | --- | --- |
| 1 | 2 | 3 | 4 |

| **Demographic Questions** |
| --- |

1. Choose one response below that best describes your experience with Inflammatory Bowel Disease:

In the last 6 months, my disease has been…(Choose one)

| Constantly active, giving me symptoms everyday | Often active, giving me symptoms most days | Sometimes active, giving me symptoms on some days | Occasionally active, giving me symptoms on some days (for instance 1-2 days/week) | Rarely active, giving me symptoms a few days in the last 6 months | I was well in the last 6 months, what I consider remission or absence of symptoms |
| --- | --- | --- | --- | --- | --- |
|  |  |  |  |  |  |

1. Have you ever been hospitalized because of your Inflammatory Bowel Disease?

Yes

No

1. Have you ever needed surgery because of your Inflammatory Bowel Disease?

Yes

No

1. What is your gender? _______________
2. What is your age? ______ years old
3. Are you of Spanish, Hispanic, or Latino descent?

Yes

No

Prefer not to answer

1. Which of the following best describes your ethnic background? (Choose one)

African American / Black

Asian / Pacific Islander

Caucasian / White

Native American

Multi-Ethnic

Other (please specify): _________________________________

Prefer not to answer

1. What is the last year of school that you completed?

8th grade or less

Some high school

High school graduate or GED

Some college or 2-year college degree

4-year college degree

More than a 4-year college degree

Prefer not to answer

1. What is your current marital or domestic status?

Married

Living with partner

Divorced or separated

Widowed

Never married

Prefer not to answer

1. We would like to get an estimate of your household income in the past 12 months before taxes. Was it… (Choose one)

Less than $5,000  $40,000 to $49,999

$5,000 to $7,499  $50,000 to $59,999

$7,500 to $9,999  $60,000 to $74,999

$10,000 to $12,499  $75,000 to $84,999

$12,500 to $14,999  $85,000 to $99,999

$15,000 to $19,999  $100,000 to $124,999

$20,000 to $24,999  $125,000 to $149,999

$25,000 to $29,999  $150,000 to $174,999

$30,000 to $34,999  $175,000 or more

$35,000 to $39,999  Prefer not to answer

1. Which of the following best describes your current employment status? (Check all that apply)

Working full time, 35 hours or more a week

Working part-time, less than 35 hours a week

Unemployed or laid off and looking for work

Unemployed, not interested in returning to work

Homemaker

In school

Retired due to pain

Retired not due to pain

Disabled, not able to work

Other (please specify): _________________________________

Prefer not to answer

1. In which branch of the military did you most recently serve? (Check all that apply)

Air Force

Army

Coast Guard

Marine Corps

Navy

Other (please specify): _________________________________

1. How long did you serve? __________ years
2. In which era(s) did you serve in the military? (Check all that apply)

World War II

Early Cold War Era (1945 to 1949)

Korean Conflict (1950 to 1953)

Cold War Era (post Korean War) (1954 to 1963)

Vietnam Era (1964 to 1975)

Late Cold War Era (1976 to 1991) (Grenada, Beirut, Libya, Panama)

Post-Cold War Era (1991 to 2001) (Persian Gulf War, Somalia, Haiti, Yugoslavia)

Post 9/11 or War on Terrorism (2001 to present)

1. What date did you complete this survey?

| _____/ | _____/ | ______ |
| --- | --- | --- |
| Month | Day | Year |

**Thank you for completing this survey!**
