# Supplementary material for: Public deliberation to assess patient views on biosimilar medication switching for the treatment of inflammatory bowel disease
Source: BMC Health Serv Res. 2024 Oct 9;24:1209. doi: 10.1186/s12913-024-11570-3 (PMC11462922; doi:10.1186/s12913-024-11570-3)
Supplement: Supplementary file 4 — Supplementary Material 4 [file 12913_2024_11570_MOESM4_ESM.docx]

Patient Preferences on Use of

Biosimilar Medications

Survey 2

**Survey Instructions**

This survey asks your opinions about treating Veterans with Inflammatory Bowel Disease. We may use this information to make recommendations to the Department of Veterans Affairs on future decisions when treating Veterans with Inflammatory Bowel Disease.

Please answer each question to the best of your ability. You may also choose not to answer any question.

| **Biosimilar Policy Rankings** |
| --- |

1. Review the five policies listed below (A-E). Rank them in order of how strongly you support them (#1 being the one you support the most, and #5 being the least). Please rank only **one** policy 1^st^, 2^nd^, 3^rd^, 4^th^, and 5^th^ choice.

| **Policy** | **Ranking** |
| --- | --- |
| **A. Status Quo:** Varies from facility to facility. Highly dependent on the individual pharmacies and physicians. New patients are often started on Biosimilars. |  |
| **B. Sickest Last:** Veterans with the least severe IBD will be switched first; Veterans with the most severe IBD, but doing well, will be switched last. |  |
| **C. Opt-Out:** Veterans will have a choice to opt-out of the switch after meeting with a pharmacist. |  |
| **D. Next Appointment:** Veterans will be switched at their next appointment after explanation from health care provider. |  |
| **E. Lottery:** Veterans will be switched based on a drawing from a lottery. |  |

1. Thinking about your ranking on the policies ….

|  | Not at all strong | Somewhat strong | Fairly  strong | Very strong |
| --- | --- | --- | --- | --- |
| 1. How strongly do you feel about your ranking? | 1 | 2 | 3 | 4 |

1. Thinking about your ranking on the policies….

|  | Not at all | Somewhat | Fairly much | Very Much |
| --- | --- | --- | --- | --- |
| 1. How much did your group discussion influence your ranking? | 1 | 2 | 3 | 4 |

1. Do you have any comments about your ranking for the policies?

| **Knowledge of Inflammatory Bowel Disease and Treatment** |
| --- |

The **purpose** of these questions is to find out what you now know about Inflammatory Bowel Disease and its treatment. You may not know all the answers and that is okay.

1. Select one answer from each row.

|  | True | False | Don’t Know |
| --- | --- | --- | --- |
| 1. There is no cure for Inflammatory Bowel Disease |  |  |  |
| 1. Irritable Bowel Syndrome is the same condition as Inflammatory Bowel Disease. |  |  |  |
| 1. Inflammatory Bowel Disease is an unpredictable disease, with periods of flares and remission. |  |  |  |
| 1. In both Ulcerative Colitis (UC) and Crohn’s Disease, the entire gastrointestinal tract can become inflamed. |  |  |  |
| 1. People can stop responding to IBD treatment over time. |  |  |  |
| 1. IBD is caused by a prolonged state of inflammation that causes injury to the gut. |  |  |  |

| **Your Views about the VA** |
| --- |

The next questions are about your opinion of the **VA Healthcare system**, in general. For each statement below, please check how strongly you agree or disagree.

|  | Strongly Disagree | Disagree | Neutral | Agree | Strongly Agree |
| --- | --- | --- | --- | --- | --- |
| - 1. The VA Healthcare System does its best to make patients’ health better. |  |  |  |  |  |
| - 1. The VA Healthcare System covers up its mistakes. |  |  |  |  |  |
| - 1. Patients receive high quality medical care from the VA Healthcare System. |  |  |  |  |  |
| - 1. The VA Healthcare System makes too many mistakes. |  |  |  |  |  |
| - 1. The VA Healthcare System puts saving money above patients’ needs. |  |  |  |  |  |
| - 1. The VA Healthcare System gives excellent medical care. |  |  |  |  |  |
| - 1. Patients get the same medical treatment from the VA Healthcare System, no matter what the patient’s race or ethnicity. |  |  |  |  |  |
| - 1. The VA Healthcare System lies to make money. |  |  |  |  |  |
| - 1. The VA Healthcare System experiments on patients without them knowing. |  |  |  |  |  |

1. The next questions are about your opinion of your **VA primary care team**. For each statement below, please check how strongly you agree or disagree

|  | Strongly Disagree | Disagree | Neutral | Agree | Strongly Agree |
| --- | --- | --- | --- | --- | --- |
| - 1. I doubt that my VA Gastroenterologist really cares about me as a person. |  |  |  |  |  |
| - 1. My VA Gastroenterology care team is usually considerate of my needs and puts them first. |  |  |  |  |  |
| - 1. I trust my VA Gastroenterology care team so much that I always try to follow their advice. |  |  |  |  |  |
| - 1. If my VA Gastroenterology care team tells me something is so, then it must be true. |  |  |  |  |  |
| - 1. I sometimes distrust my VA Gastroenterology care team’s opinion and would like a second one. |  |  |  |  |  |
| - 1. I trust my VA Gastroenterology care team’s judgement about my medical care. |  |  |  |  |  |
| - 1. I feel my VA Gastroenterology care team does not do everything they should for my medical care. |  |  |  |  |  |
| - 1. I trust my VA Gastroenterology care team to put my medical needs above all other considerations when treating my medical problems. |  |  |  |  |  |
| - 1. My VA Gastroenterology care team are experts in taking care of medical problems like mine. |  |  |  |  |  |
| - 1. I trust my VA Gastroenterology care team to tell me if a mistake was made with my treatment. |  |  |  |  |  |
| - 1. I sometimes worry that my VA Gastroenterology care team may not keep the information we discuss totally private. |  |  |  |  |  |

1. All things considered, the VA Healthcare System can be trusted

| Not true | Somewhat true | Fairly true | Very true |
| --- | --- | --- | --- |
| 1 | 2 | 3 | 4 |

| **Deliberation Day Questions** |
| --- |

**The final questions are about your views of today’s event.**

**Circle** one answer:

1. Do you feel that your opinions were respected by your group?

| Not at all |  |  |  |  |  |  |  |  | Very much |
| --- | --- | --- | --- | --- | --- | --- | --- | --- | --- |
| 1 | 2 | 3 | 4 | 5 | 6 | 7 | 8 | 9 | 10 |

1. Do you feel you were listened to by your facilitator?

| Not at all |  |  |  |  |  |  |  |  | Very much |
| --- | --- | --- | --- | --- | --- | --- | --- | --- | --- |
| 1 | 2 | 3 | 4 | 5 | 6 | 7 | 8 | 9 | 10 |

1. Do you feel that the process that led to your group’s responses was fair?

| Not at all |  |  |  |  |  |  |  |  | Very much |
| --- | --- | --- | --- | --- | --- | --- | --- | --- | --- |
| 1 | 2 | 3 | 4 | 5 | 6 | 7 | 8 | 9 | 10 |

1. How willing are you to abide by the group’s final position, even if you personally have a different view?

| Not at all |  |  |  |  |  |  |  |  | Very much |
| --- | --- | --- | --- | --- | --- | --- | --- | --- | --- |
| 1 | 2 | 3 | 4 | 5 | 6 | 7 | 8 | 9 | 10 |

1. How helpful did you find each of the following?
2. *Question and answer interaction with experts.*

| Not helpful  at all |  |  |  |  |  |  |  |  | Extremely helpful |
| --- | --- | --- | --- | --- | --- | --- | --- | --- | --- |
| 1 | 2 | 3 | 4 | 5 | 6 | 7 | 8 | 9 | 10 |

1. *The formal presentations given by the experts.*

| Not helpful  at all |  |  |  |  |  |  |  |  | Extremely helpful |
| --- | --- | --- | --- | --- | --- | --- | --- | --- | --- |
| 1 | 2 | 3 | 4 | 5 | 6 | 7 | 8 | 9 | 10 |

1. *Discussing the issues with other participants.*

| Not helpful  at all |  |  |  |  |  |  |  |  | Extremely helpful |
| --- | --- | --- | --- | --- | --- | --- | --- | --- | --- |
| 1 | 2 | 3 | 4 | 5 | 6 | 7 | 8 | 9 | 10 |

1. Overall, how would you rate the quality of the deliberative session today?

| Very low |  |  |  |  |  |  |  |  | Very high |
| --- | --- | --- | --- | --- | --- | --- | --- | --- | --- |
| 1 | 2 | 3 | 4 | 5 | 6 | 7 | 8 | 9 | 10 |

1. What device did you use to attend the Zoom meeting?

- Smart Phone
- Laptop computer
- Desktop computer
- Tablet
- Other [Please specify] ______________________

1. Was this your first time attending a Zoom meeting?

- Yes
- No

1. Did you get the right amount of information to help you prepare for the Zoom meeting?

- Too much information
- The right amount of information
- Not enough information
- No opinion

1. Have you ever attended an in-person focus group/discussion group?

- Yes
- No

1. If yes, was **listening to the presentation** easier or harder because the meeting was held virtually on Zoom?
   - Easier
   - Same
   - Harder
2. If yes, was **participating in the group discussion** easier or harder because the meeting was held virtually on Zoom?
   - Easier
   - Same
   - Harder
3. What aspects of the Zoom technology could have been improved? [Select all that apply]
   - Audio quality from device
   - Video quality from device
   - Seeing/hearing presentations
   - Seeing/hearing other participants
   - Time for discussions
   - None of the above
   - Other (please specify): ______________________________________ _________________________________________________________
4. Do you have any comments you wish to share about the session day or this study, in general?

**Thank you for completing this survey!**
