# Supplementary material for: Public deliberation to assess patient views on biosimilar medication switching for the treatment of inflammatory bowel disease
Source: BMC Health Serv Res. 2024 Oct 9;24:1209. doi: 10.1186/s12913-024-11570-3 (PMC11462922; doi:10.1186/s12913-024-11570-3)
Supplement: Supplementary file 5 — Supplementary Material 5 [file 12913_2024_11570_MOESM5_ESM.docx]

| **Appendix 5.** Exemplary quotes from small group discussions (n=29) | |
| --- | --- |
| **Theme/Domain** | **Exemplary Quote(s)** |
| **Perceived Risk of Biosimilar Switching** | As far as the difference between […] generics versus name brand or biosimilar to an originator, I think that biosimilars and originators, I’m not exactly sure what the process is for either manufacturing or growing these proteins, but I do know that if you are growing something there is always going to be a variable, nothing is ever going to be exactly the same so something that might work great for one person is not going to work as well for the other and vice versa.  When you go to the pharmacy to pick up medicine, they tell us if they're giving us Prilosec or they're just giving us Omeprazole, same drug, one Brand name, one generic, and I think it's the same thing that they should do when they give us these Biosimilars, naturally, even more so because these Biosimilars are a lot more dangerous [because] they're going straight into our bloodstream  I have a great concern that a biosimilar would not work and those symptoms would start creeping in and when they’ve indicated even more so that it’s possible that going back to the original biologic drug would not be effective anymore and that they would have to start trying the other biologics that are out there, that creates a very big, big concern for me.  …when you start feeling the symptoms you already know there's scar tissue and the scar tissue can only be resolved with surgery. It’s not a risk that I’m willing to take, because by the time you’re feeling the symptoms you know there’s scar tissue already.  I would be open to the idea about it, but I think I would be then again a little stressed and scared because you know, it takes so long to get into remission and actually feel good and to just switch it up and then possibly send me into a flare up for weeks to months, I think it’s just the thought of it is a little nerve wracking, too.  [Generics] all have the same ingredients, what are you doing? You’re paying for the name. As far as the biosimilars and the biologics I’m not really sure because I’m not very educated on them. My biggest fear is, I know everything has a risk, you take a risk by leaving your house every day. Everything you do in life has a risk. What are the benefits? We need to figure out what are the benefits of going from a biologic to a biosimilar, medically standing is it going to improve a person’s quality of life? If it does, then obviously the benefits are going to outweigh the risk. |
| **Transparency and Information** | I can’t see a reason why you would not inform, not just a Veteran, but a patient. A patient needs to be fully informed.  Participant 1: I don't think a lack of transparency would be a good thing. Participant 2: It wouldn’t be fair. It wouldn’t be something we should do.  I understand that there has to be tradeoffs, but I think the people again should be made aware of what’s going on.  Everybody should be informed on everything they’re getting. You need to know why you’re getting it, what is it doing for you, what is it, what are you going to expect, is it going to make any of your symptoms worse? Like everybody suggested before, is it something you’re doing or is it something the medication’s doing? I mean granted every medication has a long list of side effects it just, we need to know not just as Veterans but as just patients in general, they need to know what is going on with their health care, why is this going on and what is it going to do? […] Just to inform people what to expect and there’s not stress or confusion with anything.  I’d emphasize informed. I think that’s the, the basic of it all, of what would make me feel better. You know, inform me about what you’re doing to my medicine.  I believe you have to be informed because even though things are similar that means that they're not exactly the same. So that means that somebody could be allergic to something that might possibly be in the you know Biosimilar.  I think the switching can tough on a system if you’re, if it’s working, especially something like IBD I guess I think of it as differently than generic versus name brand because it is different but it, you should definitely be informed about it, if they’re thinking about it just because of that unknown, I guess.  Since it's talking about going to Biosimilar, I don't think it's ethical to change someone without telling them that you're going to change them because there is the remote risk of whatever you know, with those so if you do other changes you have to tell the patients, right? Or they have to tell us. It's not ethical to not tell.  Wouldn't you have to inform somebody of a medication change? I mean somebody is already on medicine for Crohn's or whatever, you definitely would have to inform them, right? I would want to know. And if that causes fewer resources, you’ll have to figure that one out. You definitely would have to inform somebody of a change of medicine. I mean I don't understand why you wouldn't, but we’d figure out the resources after you inform us. You know, you just can't throw a medicine on someone without telling them, and they're thinking they're taking the same thing and then all of a sudden, they have this change in their body or they have symptoms or something goes wrong and you're not informing them because you're going to have fewer resources and I don’t understand that statement.  Participant 1: I would like to know if they’re changing… that way I could manage any difference in my symptoms or anything. So, I would like to be notified and that way if there’s any changes in my progress or anything happen then we would kind of like have something to pinpoint on to go back and trace it to. Participant 2: I completely agree with Participant 1. I would want to know just in case there were any kind of changes then I would know why you know, it wasn’t something I was doing, it might’ve been the medication.  If you change our medication without us knowing and then you know, we have a little bit of difficulties, we’re going to be sitting here drilling ourselves like do I need to change my diet? What did I eat yesterday? Like what happened? What went wrong? And then you know, if weeks pass and then all of the sudden it’s like, “Oh, we changed your medicine,” I’m going to be really upset about it.  We would lose trust in both the VA and the medical field by just doing things without our knowledge. I think that would be the biggest harm to both the VA and the medical field in general. (Participant 1)  Getting the doctors to inform the patient and having a trust issue is more important than saving money because if you don’t trust the VA the Veteran’s not going to use the VA so they’ll seek healthcare somewhere else. So that’s an important part of saving money too, because it’s saving the VA from paying outside resources… (Participant 2)  If somebody is informed but doesn’t have the proper understanding to like I said, that they would freak out because they would think that it was a quality issue that they were being substituted for rather than you know, a monetary thing or something of that nature. |
| **Patient and Provider Role in Decision Making** | A person should know the risk that they’re accepting. I don’t believe that the people should be injected or given things that against their knowledge so there’s absolutely no reason why, just like the prescription tab and information sheet or the booklet, give in advance, inform the patient in advance on what you want to be changed to, that way you can make a more informed decision to see if the risks are worth accepting or not. [Later] You don’t want to risk them getting sick while you’re changing them, you want them to make an informed decision before the change, so they're not risking their health. They’re choosing to risk their health. They’re not getting their health risked by someone else.  The doctor should make the decision I think about opting out. They have so much more information than we do (Participant 1). I’d agree with that, but I’d have to say with the Veteran’s consent too, not him not knowing, you know what I’m saying? (Participant 2)  … [doctors] are the professionals, we trust in what they do and we trust that they have the skill set to tell us what's necessary for our own treatment, but at the same time I think that we do have some responsibility to understand what's going into our bodies and so with that being said I think it's absolutely vital to have that conversation… (Participant 1)  The patient does have some say. It is the patient’s life. The hospital or VA or the doctor can want to make a change for whatever other reason they have, but they still have to do it in concert with the patient. (Participant 2)  What is your life worth to you? It shouldn't be, well, I'm going to take this because it's going to save another Veteran or […] it's going to save money, there's not enough money they can put on my life so if I don't want to gamble and take a Biosimilar and I want the original I think that should be a choice. It should be a choice between you, your doctor, you guys should discuss that and if they say, well, no I want to keep the original then that's what they should do. (ID-446)I think if a Veteran is informed they have a less chance of doing the opt out so I think it would be better for the VA and being able to be cost effective if they had the doctor talking to the patient, giving him the chance yes, maybe to opt out but more persuasive in the sense that it may not be needed. See it’s the scary part in there again. Well, am I going to get sicker or you know? So, if the doctor’s talking to them, they might not get so afraid. (Participant 1)  I’m not a doctor so I would like to have that conversation with my doctor and if my doctor says, “Look, I think this is, this will work for you. Let’s give it a try,” and you know, it builds that trust and that relationship with your doctor and if it doesn’t work, it’s not a permanent solution you know, you can always go back to what you did before. I’m not opposed to saving money on me so that it can be used for another Veteran which I think every Veteran I would hope, most of us feel that way and we’re used to sharing everything, we’re not used to having the best of everything. (Participant 2)  If you ever looked on the internet lately and there’s all kinds of information out there. Not all of it’s accurate, not all of it’s good information and so again, if I was a doctor and I could diagnose myself then that would be different but one of the reasons, I think it’s key for the doctor to be the person to inform you whether it’s your primary care or your specialty doctor that builds a relationship between the doctor and the patient and I think ultimately we’re all more comfortable with somebody that we trust and it doesn’t matter if you’re a Veteran or anybody it just matters that you have a relationship that everybody trusts and you believe in the expertise of your doctor. (Participant 1)  The doctor could say, “This might be the best choice for you,” or something I mean they’ll be more familiar with the statistics than we will be, on the drugs. I firmly believe that the physicians at the VA and the (university) have […] the best, they're thinking of us all the time I think the doctors and the Veterans should closely decide this.  Well, I have to take the doctor’s words now that these biosimilars are basically the same, so I have no problem switching.  You want to be proactive but your doctor’s going to know what's best, because he already knows your medical history and everything like that so you know he's going to know what's best and what will probably work better for you. |
| **Cost and Access** | We do have to have cost cutting measures in place because there are other things that need to be purchased like perhaps may need to pay the salary of a new nurse, who knows?  We're talking about something that could be beneficial to every Veteran that has some sort of disease that requires some product and that maybe a Biosimilar’s made for and if they can do a Biosimilar for a less price then it's going to help every Veteran out.  I’m all about making sure that my fellow Veterans get access to the same resources that I do so if I can be treated with something that costs less so somebody else can be treated too, that’s good for me and as long as my doctor says that’s the best course of treatment for me. […] So I think that as long as you’re informed and your doctor says, “Yes I believe this is the best thing for you right now,” and you can have a conversation that maybe later it’ll change, what else could you want?  Maybe I’m just a little altruistic I mean if I can get the same effect and not spend as much money, even if it’s Uncle Sam’s money, I would rather get the same thing for less and I would like to think that everybody else feels the same way about that but I understand that everybody has a different philosophy when it comes to that sort of thing so I think that things should be honest and straight forward but I also think that again, we need to make sure that there is complete understanding and nobody makes knee jerk reactions.  If it worked before I don't want to be switched. I know it might be cheaper, but if it works for me now with all the problems I’ve got, I don't want someone to say let’s try a cheaper price.  It shouldn't be forced on you to go a cheaper route to save money in the end because in the end you're risking your life, you're the one that's got to pay that cost if something bad happens, you're the one that's going to pay for it so that's just my take on that.  You don’t want to just say we’re cutting this because it costs too much money and we don’t care about you.  You got to remember the Veterans and the other people first, not the money like the VA does. |
| **Personalization and Prioritization** | So, every medication has got it’s risk. Some of us react badly […] Everybody’s body is a different chemical factory, and you may react to a medication, we don’t know. The doctor doesn’t know either until he tries it. So, we will always have a risk…  Everybody's circumstances are different so for some people it can be risky and for some it may not. For me it's not risky but I know that everyone is different with their health and their circumstances.  I’m a combat medic so I learned a lot the hard way that everybody is going to react differently at different times. Something might work fine for you for one year and then next year, it doesn’t work at all so you have to have that inner connection with the system, with a doctor or a practitioner of some sort so that they can adjust things…  I have no problem with having a policy where you’re prioritizing groups of people to approach to change, that would be fine.  I feel like VA should, if they're going to change them, change them when they start at the VA there. Then wait, go back for a follow up in one to two months and see how that medicines work so they don't have to take six months of medication that isn't working.  The new patients can be offered the Biosimilars. I think that's possibly a good strategy to try it out, kind of like as a trial run you know, let's get new patients started on Biosimilars, versus the Originators.  I think you got to do it by severity. Those you think would have a lower risk of switching over, I mean, you’re going to do those first to see how it comes out and those that I think is going to have a higher effect, that it might have a chance to cause an effect, I'm going to do those last to see how the ones that I think would have little effect would work on them and then the much riskier cases, I can use that data that I collected from the easier patients to see how it would work. […] I would say those with less severe go first and those with more severe go last. |
| **Preferred Approaches**  ***“Sickest Last”***  ***Opt-out*** | …to switch a person that is really, really sick you know, I think a lot of times you know, you may lose that person, whereas you have a person that may not be as sick, where they can change medications if it doesn't work well, they can always switch back, but if you take a person that has already been through four different medications, that's had 18 different surgeries and then you going to say, “hey, look what I'm going to do is I'm going to switch you to this medication and we're going to see how that works,” to me that's just setting you up for failure.  I think the sickest being put last is the best solution because like I said, it’s part of your life, the Veteran. Yeah, it’s probably going to switch us all eventually anyway but the sickest last, at least see how it works in other Veterans and if it works well before you switch if the Veteran is really sick.  I'm a risk taker, I know but I would rather take the […] biosimilar and have someone else who’s sicker than me put right on the tried and true. [Later] The sickest person should really, really benefit from the originator drug and then after they’re stable bring them up and give them the information, tell them if they want to try it, it’s open to them to try. [Later] I still think that the sickest person should receive the best care and right now that seems to be the originator drug.  I chose sickest last first just because if in fact their situation is not as severe as someone else and this is a good alternative to the biologic then it would make sense to switch them first so that that’s more resources for other people that may need it more.  Sickest last I think makes sense in that the least affected are switched if they can be, to the biosimilar. The people having more trouble can go last which perfectly makes sense but then again in the cons […] there was no consent there with the sickest last so that’s the only reason I didn’t like that one.  I'm really strong on the sickest last and I, I just don't think you should gamble with someone’s health…  I too stand very firm on sickest last um, having been I guess, one of the sickest patients around for awhile and then to find a drug that did well and brought about a remission I think that's the last thing you want to mess with. I think you don't want to disrupt any progress or any levels of remission that you're able to achieve…  …my thinking there is that the sicker they are, the more likely they are to have a bad effect so yeah I would say the sickest last for that reason too…  I would be completely fine with sickest last because at least then I guess individuals can be switched and if there are side effects and they’re increasingly like noticeable side effects then maybe decisions can be made after that data is collected and changed from there…  I believe that the Veterans that are the sickest and are doing well on the drugs that they're on need to be switched last… [Later] if they are doing well on their medications, I just don't think they should even be switched at all they should be able to keep the same medications that they've been doing well on.  There’s an awful lot of variables that have to be taken into consideration before you can just switch somebody, but I still think that patient consent is most important.  I chose opt out as one is because it’s supporting the Veterans freedom the most and after all the VA is serving the Veteran so that’s why I voted that way.  I definitely think it's important you know, the opt out even though our doctor knows the best care for us, we still need an opinion as well. Our voice needs to be heard whether we want to take it or not…  Where's the data? How much of its working? I need to see some data. I mean I'm still terrified about this. You know I want the option not to do it  I do believe if you can make an informed decision on the medication and the side effects that you should have the right to opt out. However, that's dependent on talking to the right people. You can't just opt out because you say, “I don't want this in my system. I don't know what's in it.” How many of us know what's in every medication we take anyway?  A lot of people if they have the choice not to change, if they’re doing good, they’re going to want to stay with the medicine they’re on they’re not going to want to try something like the VA wants them to so it’s not going to be any cost effective, as cost effective as just switching everybody.  Opt out? I don't believe opt out would work. You can't have people, there's millions of us, if everybody decides to do something separate, we've already seen the examples in the Washington of what happens when people get to have their own opinion, so it's not a pretty thing to have so I would never allow an opt out. [Later] The opt out. I don't think that should ever be an option. You can't get anything done. If you make people say I don't want to play, you'd have a hell of a baseball game.  I chose opt out as the first choice because that basically gives the Veteran the most choice of what all he has to do and the problem with that might be that maybe the Vet is just choosing the most expensive drug out there because he doesn't want to have a switch or doesn't want to change at all, doesn't want anything to do with these Biosimilars and that's not really good for the VA...  I think that all the information should be given to the patient and the patient give the option to opt out or stay with the, the originator.  I think that you know, opt out has to be a part of that next appointment conversation. I think the two should be combined.  I would […] keep my opt out as number one with consent after speaking with the health care provider and the pharmacist. [Later] Veteran who’s put in their time, can’t say no to something, I don't think that’s right so.  … you shouldn’t be forced into changing medication or doing any procedure you don’t feel comfortable doing.  I chose opt out as my first choice just because I think, I think between you and your doctor I think each of you can make the best decision on whether it's in your best interest to switch to a Biosimilar or stick with the Originator. So I think with all the information you know, your physician can actually offer you, you should be, at that point being able to make a you know, a good decision whether to switch or not so that's why I chose opt out as number one for me.  … it does give the Veteran you know, some empowerment in the decision but based on the best information possible. |
| ***Less Preferred Approaches***  ***Next Appointment***  ***Status Quo***  ***Lottery*** | … if you’re saying that you couldn’t say no at the next appointment then I would not put that as number one so I would have to go back and keep my opt out as number one with the consent you know, with consent after speaking with the primary or the health care provider and the pharmacist  It seems to me that if you're on your next appointment and you feel very strongly that the Biosimilar was not going to work for you and you're very determined that you want to keep the one you have, you should be able to make that clear to your doctor at that time…  I picked as my number one next appointment because that's really when you can get all of your questions answered by your physician…  I don't like just the idea of just uh, doing it at the next appointment and stuff like that so I feel that the Veteran should always be informed…  … if next appointment is sporadic you know, it could be six months, it could be year, could be a year and a half. These appointments, these next appointments don't have a solid date you know what I mean? So I mean, when is that next appointment going to be?  … it should be done in the presence of a health care provider so that they’re able to explain to them what’s going on and also I think doctors should have the ability to say, “For this particular patient, no, we’re not going to do that.”  I chose next appointment was because I was considering the conversation, the dialogue that would take place between the patient and the doctor in order to maximize the benefit of the drug while at the same time being impactful in a financial way.  … at their next appointment have a conversation, an in-depth conversation about the biosimilars and inform the Veteran you know, the doctor could say, “This might be the best choice for you.”  {At the next appointment] the doctor can talk to you and explain and then make a logical decision on your health and I guess that's the main thing is that I prefer making my own decisions if I can do it but you have a doctor there supposedly who can answer these things.  My most preferred was next appointment. I just kind of seems to be where it should happen when you are talking with your doctor about what's going on and why they want to switch you.  The only part that you know, was not good about that is that it’s based on your region or your state or whatever. It should be across the VA system […] When I retire [and] become a snow bird and go to Florida that would be awesome or Arizona or whichever, but I want have my same options in any state I decide to go to  The status quo right now is people are getting switched and they don’t even know it.  I mean I've been in three different VA systems […] what if I start going back to (City) and they don't have what I need? Am I going to have to fly all the way back to (City) just to get what I need? You know, so I didn't think that that was fair for, not only for myself but for Veterans nationwide like I think it should be the same across the board.  I would just prefer it to be a common, coherent and coordinated policy, not kind of disjointed across various areas.  I would say I chose lottery as the last because it doesn't take into effect any other things like it doesn't take into effect severity, it doesn't take into effect no, no other knowledge, it's just basically put everybody names in a hat and however it falls, fall and basically that's the equivalent of pretty much, almost doing nothing.  Particularly the lottery, I mean nothing that involves somebody’s life should ever be based on the roll of a dice. [Later] If it just happens to be done with a lottery like that and your number comes up, who bears the culpability if something bad happens as a result of that decision?  If I am severely sick I wouldn't want to be thrown into a lottery pool.  My least preferred I put down as the lottery system. I don't play lottery tickets. I don't think a lottery is in anywhere, it's nothing but the luck of the draw and I think we've heard enough about that to understand that's really no treatment plan at all. I think treatment medication should be gauged towards the patient not just if my number comes up or not.  I think a lottery is the worse option because I would hate to think that I got something that somebody else needed worse than I do…  I don't think a individual’s health should be put on hold or be left up to a lottery type decision.  … lottery it’s not informing anyone, it’s just giving everyone a number and you’re being switched automatically it’s just a matter of when you do get switched it’s randomized.  As far as the lottery option I mean there's lotteries that you hope to win and there's lotteries that you don't hope to win… |
